# The Evolution of Imperfection: Review of Hurst, L.D., 2025, *The Evolution of Imperfection: The Science of Why We Aren’t and Can’t Be Perfect Publisher*, Princeton University Press. Price: $29.95/£25.00, Princeton, NJ, 288 pages. ISBN: 9780691247397.

**DOI:** 10.1093/emph/eoaf042

**Published:** 2026-01-03

**Authors:** Norman A Johnson

**Affiliations:** Department of Biology, UMASS Amherst, Amherst, MA 01003, United States

The coverage of evolution in textbooks and popular books alike usually emphasizes its triumphs—look at the hummingbird flapping its wings extraordinarily quickly; look at the fit between the bills of Darwin’s finches and the seeds they eat; look at the fit between the colors of moths and the color of trees on which they rest. This is all well and good; these successes make for excellent stories. But there another side of evolution: its failures and its wastefulness. Laurence Hurst takes us on a journey through this darker, more dilapidated side of evolution in his recent book, *The Evolution of Imperfection*. Hurst, an evolutionary geneticist at the University of Bath, is a gifted story teller and is well-equipped as our tour guide.

Why a book on evolution’s imperfections? There are at least two compelling reasons. First, discussion of its imperfections helps combat the pernicious idea of evolution as a “march of progress.” This false idea is often seen in cartoons depicting evolution, wherein a bent-over ape becomes a fully-erect human over a few steps. Aside from being inaccurate, the march of progress also reinforces the notion that humans are its pinnacle. Most troublingly, it can promote social Darwinism, the ugly idea that evolution justifies hierarchies among groups of people. Showing evolution’s flaws and foibles helps combat these harmful misconceptions. Second, and of particular relevance for the readership of this journal, these imperfections of evolution underlie much of why we get sick [1]. Hurst reminds us that medicine often is a struggle against nature, noting: “it is our best attempt to stop nature from taking its course, whether it be by curing kids of cancer, taking antibiotics to fend off bacterial infections, swapping out bad hearts for good ones, or overcoming infertility with in vitro fertilization (IVF).” (p. 11). Just as medicine is the practice of combating the ills caused by nature, evolutionary medicine is the science of understanding why nature—that is, evolution—causes these ills. We gain insight into disease by examining why evolution is imperfect; with this insight, an evolutionary perspective can inform medical practice in treating disease, a theme Hurst explores in depth at the end of the book.

Hurst is not the first to address malfunction brought on by evolution’s imperfections. Prior authors, however, typically focused on anatomical imperfections resulting from the legacies of our evolutionary history [2]. These failings include the literal blind spots vertebrate eye evolution has given us, the choking risks brought on by the use of the same machinery for respiration and digestion, and the back aliments resulting from bipedalism. Hurst, instead, emphasizes imperfections that manifest at the genome level. He is particularly interested in those arising from two major causes: the accumulation of deleterious mutations and conflict between different parts of our genome that differ in their evolutionary interests.

Mutation is an important, and often misunderstood, evolutionary process. First, although the probability of a mutation in any one individual site in the genome is exceedingly rare, every person has dozens or more new mutations because the human genome is large. We are—each of us—all mutants. Second, most of these mutations are deleterious: some have catastrophic effects, while others have more subtle effects or are deleterious only in some environments. Moreover, most of these mutations are shielded because their effects are recessive (manifesting mainly in homozygotes). This is why inbreeding often results in deleterious effects. Despite usually having deleterious effects, mutations sometimes are advantageous, either globally or in some contexts.

As Hurst points out, recurrent mutation is the primary reason that rare single-gene diseases persist in the population. Even though selection reduces the frequency of the disease-carrying allele, mutation continually brings more copies. The result is that the allele is maintained at a low frequency: u/s, if the allele is dominant, and the square root of that quotient, if it is recessive. Given that mutation rates are typically many orders of magnitude lower than selection coefficients, alleles maintained this way are typically rare. But humans have lots of essential genes. Accordingly, even though rare genetic diseases are individually rare by definition, rare diseases collectively are very much not rare. Hurst suggests that, combined, rare diseases affect 5%–10% of us.

Disease-causing genes are under purifying selection; and thus, should evolve slower than the average gene. Hurst does point out a subtle feature: disease-causing genes evolve slowly, but are not extreme laggards in their rates of evolution. As he reminds us: “A genetic disease is defined in terms of humans that have survived at least to birth. If a gene and all its parts are so vital that mutations kill you as a very early embryo, then we never get to see these, and they don’t get classified as disease-causing mutations.” (p. 82).

Two other key points Hurst makes are that our mutation rate is substantially higher than its expected optimum and our genomes are bloated. Both features arise from the same source: the low long-term effective population size of our lineage. Because our mammalian and earlier ancestors have gone through periodic bottlenecks, our effective population size is many orders of magnitude smaller than our census population size. Accordingly, natural selection has not been as efficient in weeding out slightly deleterious variants in our lineages than it has those of insects and especially microbes. As a consequence, over the eons, our genomes have accumulated baggage. Moreover, our mutation rate, which is itself subject to evolutionary processes, is higher than theory would suggest is optimal. This feature leads to more genetic disease, as highlighted in the preceding paragraphs. This idea, known as the drift-barrier hypothesis, is well known within population-genetic circles, but not as much outside of those circles. It serves as a reminder that natural selection is not the only evolutionary process. Echoing Dobzhansky [3] and Lynch [4], Hurst shows that little in evolutionary medicine makes sense except in the light of population genetics.

Not all of our ills are due to the rain of deleterious mutations. Another source is the genomic conflict that arises because different parts of genomes differ in their evolutionary interests. For instance, a mitochondrial variant that enhanced the fitness of its female carriers at the expense of its male carriers, would be favored because males are dead-ends for mitochondria, which are only transmitted through females. At the extreme are selfish genetic elements; these molecular parasites increase their transmission at the expense of the host. Early in his career, Hurst proposed a provocative—and in retrospect, prescient—hypothesis that the evolution of selfish genetic elements and their repressers were major contributors to hybrid incompatibility, an important component of speciation [5]. At the time, this hypothesis generated much commotion, even scorn. During the ensuing decades, accumulating evidence has shown that Hurst’s hypothesis was right: much of hybrid incompatibility arose as remnants of genomic battles involving selfish genetic elements [6–8].

Hurst was attracted to the study of genomic conflict because of the “intellectually delicious” evolutionary problems it poses. As he notes, they “challenge just about every presumption you might have had about evolution and natural selection” (p. 189). But genomic conflict and selfish genetic elements are more than mere intellectual curiosities; they pervade biology and are highly relevant to evolutionary medicine. For instance, consider how sperm develop. There is tremendous evolutionary pressure for the precursors to sperm, sperm stem cells, to “cheat”: those that become sperm have a chance to get to pass on genes to the next generation while those that do not become sperm are evolutionary dead-ends. Thus, alleles that increase the probability that a stem cell will become sperm have an evolutionary advantage even if may be detrimental to the organism. Hurst suggests some disorders such as Noonan syndrome and achondroplasia (a form of dwarfism where short stature is due to having very short limbs) as possible cases of this phenomenon. In these putative cases of diseases arising from conflict among sperm stem cells, the mutation typically comes from the father, as expected.

Pregnancy is another source of genomic conflict. A fetus has alleles that come from the father and those that come from the mother. These alleles have different evolutionary interests: the paternal alleles benefit primarily from the success of the focal fetus, while the maternal alleles also need to consider the other offspring (current and future) of the mother. Accordingly, the optimal amount of resources that the fetus should take from the mother is greater for the paternal alleles than it is for maternal alleles. This conflict occasionally manifests in preeclampsia and other health problems in pregnancy. This evolutionary view of pregnancy has been developed mostly by Haig [9] and his associates. It rests upon the assumption that, over the course of the evolutionary history of our lineage, offspring from the same mother often have different fathers. This sounds very reasonable. One prediction of Haig’s model that recently has been borne out is that pregnancy is more risky for mothers when the fetus and the mother are not genetically related, such as in surrogacy [10].

Hurst’s message, which he expresses in the final chapter, is that against the tyranny of mutation and natural selection, we have medicine (and public health). He starts the chapter with noting that the British Medical Journal polled its readers in 2007 regarding the greatest medical triumphs since 1840 (the start of the journal). In order from the most important down, the answers are: Clean water/sanitation, antibiotics, anesthesia, vaccines, and then the structure of DNA. Hurst notes that the germ theory of disease went along with improved sanitation in the later half of 19th century United Kingdom. Hurst notes that much of the improved lifespan and health across the world in the last few centuries has come from these apparently simple measures. Unfortunately, a depressingly large segment of the American public circa 2025 resists vaccines and to an extent, germ theory. Regarding genomic medicine, Hurst states that while he’s in favor of it, he also concedes “that the dawn of genomic medicine remains a rather slow dawn: the sun is rising, just not all that fast.” (p. 227). He notes that less costly and less sophisticated measures often can be successful. For instance, he notes that the mass provisioning of mosquito nets across Africa has led to a dramatic decline in deaths from malaria.

The Evolution of Imperfection comes close to perfection. I highly recommend it to those from medicine who are interested in a thoughtful and nuanced discussion of the relevance of evolutionary genetics to their practice. I also recommend it to biologists interested in evolutionary medicine. It will provide much background reading for evolutionary medicine courses.
